# Risk factors for intestinal metaplasia in concomitant gastric and duodenal ulcer disease

**DOI:** 10.3892/etm.2014.1507

**Published:** 2014-01-28

**Authors:** JUN-BO HONG, LIANG XIA, WEI ZUO, AN-JIANG WANG, SHAN XU, HUI-FANG XIONG, YOU-XIANG CHEN, XUAN ZHU, NONG-HUA LU

**Affiliations:** 1 Department of Gastroenterology, The First Affiliated Hospital of Nanchang University, Nanchang, Jiangxi 330006, P.R. China; 2 Department of Respiratory Medicine, The First Affiliated Hospital of Nanchang University, Nanchang, Jiangxi 330006, P.R. China; 3 Department of Pathology, The First Affiliated Hospital of Nanchang University, Nanchang, Jiangxi 330006, P.R. China

**Keywords:** *Helicobacter pylori* infection, risk factors, concomitant gastric and duodenal ulcers, intestinal metaplasia

## Abstract

The aim of this study was to estimate the prevalence and risk factors of intestinal metaplasia (IM) in concomitant gastric and duodenal ulcer (CGDU) disease by retrospectively reviewing consecutive patients who had undergone esophagogastroduodenal endoscopy. Patients who received the endoscopic diagnosis of CGDU disease were selected for analysis and the recorded demographic, endoscopic, clinical and outcome data, including data on the development of IM, were extracted. Associations of the various parameters with IM were estimated by logistic regression analysis and described by the odds ratio (OR) with a 95% confidence interval (CI). Among the total 204,073 consecutive patients screened, 2,397 (1.2%) were diagnosed with CGDU disease. Following application of the exclusion criteria, a total of 2,149 cases were included in the study. The IM prevalence was 8.4%, represented by 153 mild cases, 26 moderate cases and one severe case. Multivariate analysis identified age ≥50 years (OR=2.606, 95% CI=1.889–3.597, χ^2^=34.000, P<0.001), ulcer at the gastric incisura (OR=2.644, 95% CI=1.926–3.630, χ^2^=36.142, P<0.001) and *Helicobacter pylori* (*H. pylori)* infection (OR=2.338, 95% CI=1.573–3.474, χ^2^=17.648, P<0.001) as independent risk factors for the development of IM. In addition, the moderate and severe IM grades were more frequently detected in males than in females (18.8% vs. 5.8%; OR=3.769, 95% CI=1.083–13.121, χ^2^=4.887, P=0.036). IM in patients with CGDU disease is not uncommon. CGDU patients with ongoing *H. pylori* infection, gastric incisura involvement, older age and/or male gender may be at a higher risk of IM.

## Introduction

Gastric cancer (GC), the second and fourth most common type of cancer in males and females respectively, is one of the leading causes of cancer mortalities worldwide (1–5). The intestinal type of GC develops through a cascade of well-defined and recognizable precursors [inflammation-atrophy-intestinal metaplasia (IM)-dysplasia-carcinoma sequence] (6). The annual incidence of GC within five years after diagnosis is appreciable in patients with atrophic gastritis (0.1%), IM (0.25%), mild-to-moderate dysplasia (0.6%) and severe dysplasia (6.0%) (7). In addition, GC development has been reported to occur >10.9-fold more frequently in the presence of IM (8). These findings indicate that IM is an important turning point in the development of GC. Thus, risk factors that are closely associated with IM should be determined in order to monitor or prevent GC onset.

Peptic ulcer disease (PUD), including gastric ulcers (GUs) and duodenal ulcers (DUs), is mainly caused by *Helicobacter pylori* (*H. pylori)* infection (9–11). Previous studies have characterized GUs as a precancerous condition of GC (12), shown that a past history of GU confers an increased risk of GC (13), and revealed a high frequency of IM detection in the GU patient population (14), indicating that IM is a key pathology that requires particular attention in the presence of GUs. However, DUs are rarely associated with IM, and thus are considered to play a minimal role in the development of GC (15,16). Furthermore, a study indicates that factors associated with DUs may be protective against GC (17). However, patients with DUs have an increased risk of GC in the presence of IM at the antrum and corpus lesser curvature, and thus those patients with DUs should be screened for IM and followed-up for GC development (18).

Concomitant gastric and duodenal ulcer (CGDU) disease, which is defined as the co-existence of ulcers in the stomach and duodenum, is a special type of PUD and is clinically characterized by the predominant symptoms of epigastric pain accompanied by bloating, acid reflux/regurgitation, bleeding, nausea, vomiting, poor appetite, early satiety and heartburn (10,19). However, the prevalence of IM in patients with CGDU disease has not been fully elucidated by studies using a large number of patients. Additionally, the potential risk factors for IM present in patients with CGDU disease require identification, as does the prevalence of *H. pylori* infection and the pathological characteristics in Chinese patients with CGDU disease.

Therefore, the aim of the present study was to determine the prevalence of IM and the associated risk factors in Chinese patients with CGDU disease.

## Patients and methods

### Inclusion and exclusion criteria

The medical records of consecutive patients who underwent esophagogastroduodenal endoscopy due to upper gastrointestinal symptoms between January 2002 and August 2011 at the First Affiliated Hospital of Nanchang University (Nanchang, China) were retrospectively reviewed, and the patients who were diagnosed with CGDU disease were selected for further analysis. The study was approved by Ethics Committee of the First Affiliated Hospital of Nanchang University and informed consent was obtained from each individual. The upper gastrointestinal symptoms included epigastric pain, bloating, acid reflux, bleeding, nausea, vomiting, poor appetite, early satiety, and heartburn. Patients who had a history of anti-*H. pylori* therapy, or who had been treated with non-steroidal anti-inflammatory drugs (NSAIDs) over the prior three months or with histamine H_2_-receptor antagonists or proton pump inhibitors over the prior four weeks were excluded.

At endoscopy, an ulcer was defined as a deep mucosal break with a diameter of ≥5 mm in the stomach or duodenum (for active ulcers) (10), or by evidence of scarring or deformity. CGDU disease was diagnosed when there were ulcers in the stomach and duodenum, with or without complications (i.e., gastrointestinal bleeding and perforation). Cases with histologically confirmed GC and dysplasia were excluded.

As numerous patients had more than one endoscopy performed during the period of study, the first endoscopy was used as the index. However, when a patient had CGDU disease and/or IM, the first endoscopy at which the CGDU disease and/or IM was diagnosed was used as the index. Thus, each patient had one representative endoscopy (20). In addition, as a number of patients had more than one ulcer in the same and/or different sites of stomach or duodenum, the ulcer with the largest diameter was considered as the index ulcer; for these cases, the size of the GU or DU was included in the analysis.

### Diagnosis of H. pylori infection and upper gastrointestinal symptoms

At the time of endoscopic examination, biopsies were obtained from the antrum (n=2) and body mucosa (n=2) without the ulcer, and the edge of each ulcer (n≥2). One antral biopsy was used for an in-house rapid urease test (RUT), and the remaining biopsies were used for histological examinations and Giemsa staining. *H. pylori* infection was defined if the RUT, histological examination or Giemsa staining was positive.

The presence of upper gastrointestinal symptoms characteristic of CGDU (10,19), including epigastric pain, bloating, acid reflux/regurgitation, bleeding, nausea, vomiting, poor appetite, early satiety and heartburn, was recorded.

### Histological examinations

Histological examinations were performed by experienced pathologists at the First Affiliated Hospital of Nanchang University. Histological changes, including chronic and active inflammation, lymphoid aggregates or follicles, atrophy, and, in particular, IM and grading, were determined according to the updated Sydney System (21).

### Statistical methods

Data are presented as the mean ± standard deviation (SD) or percentage rate. The t-test (for numerical data) and the χ^2^-test or Fisher’s exact test (for categorical data) were used to estimate the significance of the differences, which were described by the odds ratio (OR) and 95% confidence interval (CI). Variables with P<0.10 in the univariate analysis were entered as candidate risk factors in the multivariate forward stepwise logistic regression analysis. All statistical analyses were performed by the Statistical Package for Social Science software suite (version 17.0; SPSS, Inc., Chicago, IL, USA). P<0.05 was considered to indicate a statistically significant difference, and all reported P-values were two-sided.

## Results

### Prevalence and endoscopic findings of CGDUs in patients undergoing oesophagogastroduodenoscopy endoscopy

Among the 204,073 consecutive patients who underwent esophagogastroduodenal endoscopy with a defined index endoscopic procedure, 2,397 patients were diagnosed with CGDU disease, which accounted for 1.2% of the overall cases. Of these 2,397 patients, 248 were excluded. The reasons for exclusion were GC (n=11), dysplasia (n=15), a history of anti-*H. pylori* therapy (n=43), or treatment with NSAIDs, H_2_-receptor antagonists or proton pump inhibitors (n=179). Thus, 2,149 cases (1,610 males and 539 females, with a mean age ± SD of 46.0±13.5 years) with CGDU disease were included in the study. Of these cases, index GUs in the gastric antrum, gastric incisura and gastric corpus (including the gastric fundus and cardia) were present in 1,367, 718 and 64 cases, respectively, and index DUs in the superior, inferior, anterior and posterior walls were present in 489, 436, 1,031 and 193 cases, respectively. Two or more GUs and DUs were found in 279 and 312 patients, respectively. Gastrointestinal bleeding and perforation occurred in 216 and 19 patients, respectively. The mean sizes of the index GUs and DUs were 5.7±2.7 mm and 7.2±3.4 mm, respectively (Table I).

### Symptomatic pattern, H. pylori infection and IM in patients with CGDU disease

The predominant symptom of the patients with CGDU disease was epigastric pain (77.2%) accompanied by other symptoms, including bloating (57.1%), acid reflux/regurgitation (48.8%), bleeding (10.0%), nausea (34.3%), vomiting (9.4%), poor appetite (34.6%), early satiety (11.3%) and heartburn (31.3%) (Table II).

Of the 2,149 patients with CGDU disease, 1,486 (69.1%) patients were positive for RUT, histological examination and/or Giemsa staining, and thus defined as having an ongoing *H. pylori* infection (Table II). IM was observed in 180 (8.4%) patients, of which 128 (71.1%) were male and 52 (28.9%) were female, and 153 (85.0%) showed mild grade, 26 (14.4%) showed moderate grade and one (0.6%) showed severe grade IM (Table II).

### Correlations of demographic, clinical and endoscopic characteristics and H. pylori infection with IM

Patients with IM were significantly older than those without IM (52.4±11.8 vs. 45.4±13.6 years; P<0.001). However, no significant difference was identified in the incidence of IM between the males and females (8.0 vs. 9.6%; OR=0.809, 95% CI=0.577–1.134, χ^2^=1.516, P=0.218). No differences were found in the rates of IM that occurred for any of the symptoms, nor between different sizes of GU or DU (P>0.05). *H. pylori* infection was detected in 81.7% of the patients with IM, which was significantly higher than the incidence (68.0%) in the patients without IM (OR=2.096, 95% CI=1.420–3.093, χ^2^=14.431, P<0.001; Table III). Also, GUs at the gastric incisura were associated with an increased rate of IM, compared with the rate of IM associated with GUs at other locations (56.7 vs. 43.3%; OR=2.872, 95% CI=2.107–3.915, χ^2^=47.757, P<0.001). Multivariate analysis identified age ≥50 years (OR=2.606, 95% CI=1.889–3.597, χ^2^=34.000, P<0.001), GUs at the gastric incisura (OR=2.644, 95% CI=1.926–3.630, χ^2^=36.142, P<0.001) and *H. pylori* infection (OR=2.338, 95% CI=1.573–3.474, χ^2^=17.648, P<0.001) as independent risk factors for the development of IM (Table III).

In addition, the mean age of the patients with moderate and severe IM was 56.0±10.1 years, which was slightly older than that of the patients with mild IM (51.8±12.0) (P=0.091). Moderate and severe IM were more frequently detected in males than in females (18.8% vs. 5.8%; OR=3.769, 95% CI=1.083–13.121, χ^2^=4.887, P=0.036) (data not shown).

## Discussion

The prevalence of PUD, diagnosed predominately by endoscopy (22), has been reported as 4.1% in the general adult population of northern Sweden (23), and 2.7% in Italian individuals without evidence of *H. pylori* infection or prior exposure to NSAIDs (10). In the USA, the prevalence of GUs, DUs and CGDUs has been reported to be 3, 6 and 0.8%, respectively (24). In China, the endoscopic detection rate of PUD has been reported as 17.2% (25). However, the prevalence of CGDU disease has not been considered in these studies, but was calculated as 1.2% in the population of Chinese patients in the present study.

*H. pylori* infection and NSAIDs and/or aspirin use remain the main determinants of PUD (9,11); the ultimate indication that *H. pylori* infection is the main cause of PUD is the permanent cure of ulcers upon eradication of the infection (26,27). In addition, early *H. pylori* eradication is also associated with a decreased risk of GC in patients with PUD (28). Therefore, it is critical to identify *H. pylori* infection in patients with PUD. A previous study reported that the prevalence of *H. pylori* infection was 77.6% in bleeding PUD in Japanese patients (29). Another study conducted in Poland detected *H. pylori* infection in 72.5% of GU patients, in 83.6% of DU patients and in 76.9% of CGDU patients (30). However, the prevalence of *H. pylori* infection in Chinese patients with CGDU disease has been poorly reported. In the present study, the prevalence of *H. pylori* infection in a large cohort of patients with CGDU disease was found to be 69.1%. Based on the findings of a previous study (31), eradication treatment for *H. pylori* infection should be adopted to heal ulcers and reduce relapse.

The role of *H. pylori* infection in the development of IM in patients with CGDU disease has not been well elucidated. The present study observed that the prevalence of *H. pylori* infection in CGDU disease patients with and without IM was 81.7% and 68.0%, respectively, and multivariate analysis showed that *H. pylori* infection was an independent risk factor for IM in patients with CGDU disease. This indicates that *H. pylori* infection must be eradicated in these patients for a new reason - prevention of the development of IM and thus termination of the progression to neoplasia (32), which ultimately hinders the occurrence of GC.

The associations of age and gender with IM in patients with CGDU disease have, to the best of our knowledge, not been reported previously. In the present study, multivariate analysis revealed that age ≥50 years was an independent risk factor for IM, which is consistent with previous studies reporting that the incidence of IM increases with age (8,33). In addition, although the overall incidence of IM between the male and female patients was not observed to be significantly different, moderate and severe IM occurred more commonly in males than in females, suggesting a high degree of IM in male patients, which is prone to developing into GC. These findings further supported the theory that increased age and male gender were risk factors for the development and increasing severity of premalignant gastric lesions, including IM, and thus for the development of GC (7).

IM is often unevenly distributed throughout the stomach (32), and occurs most frequently along the lesser curvature from the cardia to the pylorus, particularly in the transitional zones (mainly from the corpus to the antrum) (34). A previous study revealed that the prevalence of IM was higher in the antral mucosa than in the body mucosa, and the highest frequency of IM was detected in patients with GUs (14). Thus, the specific ulcer sites that are closely associated with the development of IM should be determined, particularly in patients with CGDU disease. In the present study, multivariate analysis identified that ulceration at the gastric incisura was an independent risk factor for IM. In addition, no difference was identified in the rates of IM among the ulcers at different duodenal sites. These findings suggest that ulcers at the gastric incisura are closely associated with the development of IM. Notably, in the present study, no association was identified between any of the symptoms, as well as the size of the ulcers, with IM, indicating that these two factors were not predictive factors for IM.

The present study has certain limitations that may affect the interpretation of the findings. Sub-analyses were not performed for the subtypes of IM [complete vs. incomplete IM, the latter of which has been suggested as a risk factor for GC development (32)]. The difference in the prevalence of IM between CGDUs and GUs alone was not evaluated in the present study. Additionally, this was a retrospective study, and thus there was no regular follow-up of the patients; follow-up is essential to determine the long-term histological consequences (i.e., dysplasia and GC) of patients with CGDU disease and IM. Therefore, the clinical significance of the risk factors that were identified to be associated with IM in patients with CGDU disease requires verification in long-term prospective studies.

In conclusion, CGDUs were observed in 1.2% of the patients in the single-site Chinese population of the present study. IM occurred in 8.4% of those patients with CGDU disease. *H. pylori* infection, age ≥50 years and ulceration at gastric incisura were identified as independent risk factors for IM in patients with CGDU disease, whereas males were observed to be more prone to moderate/severe IM than females. Further long-term prospective investigation is required to verify these findings.

## Figures and Tables

**Table I:** Endoscopic findings of patients with CGDU disease.

Parameter	Measure
Exclusion (n)	
GC	11
Dysplasia	15
History of anti-*H. pylori* therapy	43
Treated with NSAIDs, H_2_-receptor antagonists or proton pump inhibitors	179
Inclusion (n)	
Benign CGDU	2149
Gender (n)	
Male	1610
Female	539
Site of index GU^a^ (n)	
Gastric antrum	1367
Gastric incisura	718
Gastric corpus^b^	64
Site of index DU^a^ (n)	
Superior wall	489
Inferior wall	436
Anterior wall	1031
Posterior wall	193
Complications (n)	
Gastrointestinal bleeding	216
Perforation	19
Size of CGDU (mm ± SD)	
GU	5.7±2.7
DU	7.2±3.4

a 591 patients had GUs at two or more sites within the stomach (n=279) or at two or more sites within the duodenum (n=312).

b Including four cases in the gastric fundus and one in the cardia, all without IM.

CGDU, concomitant gastric and duodenal ulcer; GC, gastric cancer; *H. pylori, Helicobacter pylori*; NSAIDs, non-steroidal anti-inflammatory drugs; GU, gastric ulcer; DU, duodenal ulcer; IM, intestinal metaplasia.

**Table II:** Symptomatic pattern, *H. pylori* infection and IM in 2,149 patients with CGDU disease.

Parameter	Percentage
Upper gastrointestinal symptom^a^	
Epigastric pain (n=1660)	77.2
Bloating (n=1228)	57.1
Acid reflux/regurgitation (n = 1050)	48.8
Bleeding (n=216)	10.0
Nausea (n=737)	34.3
Vomiting (n=202)	9.4
Poor appetite (n=744)	34.6
Early satiety (n=243)	11.3
Heartburn (n=673)	31.3
*H. pylori* status	
Positive (n=1486)	69.1
Negative (n=663)	30.9
IM	
Presence (n=180)	8.4
Gender	
Male (n=128)	71.1
Female (n=52)	28.9
Extent	
Moderate/severe (n=27)	15.0
Mild (n=153)	85.0
Absence (n=1969)	91.6

a Certain patients had two or more symptoms.

*H. pylori, Helicobacter pylori;* IM, intestinal metaplasia; CGDU, concomitant gastric and duodenal ulcer.

**Table III:** Correlation of demographic, clinical and endoscopic characteristics and *H. pylori* infection with IM.

Parameter	With IM n=180	Without IM n=1969	Univariate analysis		Multivariate analysis	
	
			OR (95% CI)	P	OR (95% CI)	P
Age in years						
≥50	108 (60.0%)	700 (35.6%)	2.719 (1.990–3.716)	<0.001	2.606 (1.889–3.597)	<0.001
<50	72 (40.0%)	1269 (64.4%)				
Gender						
Male	128 (71.1%)	1482 (75.3%)	0.809 (0.577–1.134)	0.218		
Female	52 (28.9%)	487 (24.7%)				
Upper GI symptoms						
Epigastric pain	133 (73.9%)	1527 (77.6%)	0.819 (0.578–1.161)	0.262		
Bloating	107 (59.4%)	1121 (56.9%)	1.109 (0.813–1.513)	0.514		
Sour regurgitation	82 (45.6%)	968 (49.2%)	0.865 (0.637–1.175)	0.354		
Bleeding	20 (11.1%)	196 (10.0%)	1.131 (0.694–1.841)	0.621		
Nausea	58 (32.2%)	679 (34.5%)	0.903 (0.652–1.251)	0.541		
Vomiting	18 (10.0%)	184 (9.3%)	1.078 (0.647–1.795)	0.773		
Poor appetite	66 (36.7%)	678 (34.4%)	1.103 (0.803–1.515)	0.543		
Early satiety	25 (13.9%)	218 (11.1%)	1.296 (0.830–2.022)	0.253		
Heartburn	61 (33.9%)	612 (31.1%)	1.137 (0.823–1.570)	0.437		
Site of index GU^a^						
Gastric antrum	76 (42.2%)	1291 (65.6%)	0.384 (0.281–0.523)	<0.001		
Gastric incisura	102 (56.7%)	616 (31.3%)	2.872 (2.107–3.915)	<0.001	2.644 (1.926–3.630)	<0.001
Gastric corpus^b^	2 (1.1%)	62 (3.1%)	0.346 (0.084–1.425)	0.166		
Site of index DU^a^						
Superior wall	35 (19.4%)	454 (23.1%)	0.805 (0.549–1.182)	0.268		
Inferior wall	30 (16.7%)	406 (20.6%)	0.770 (0.513–1.157)	0.207		
Anterior wall	96 (53.3%)	935 (47.5%)	1.264 (0.931–1.716)	0.133		
Posterior wall	19 (10.6%)	174 (8.8%)	1.217 (0.738–2.008)	0.440		
Size of GU in mm	5.6±2.9	5.7±2.7		0.469		
Size of DU in mm	7.3±3.9	7.1±3.3		0.469		
*H. pylori* infection	147 (81.7%)	1339 (68.0%)	2.096 (1.420–3.093)	<0.001	2.338 (1.573–3.474)	<0.001

a 591 patients had GUs at two or more sites within the stomach (n=279) or at two or more sites within the duodenum (n=312).

b Including four cases in the gastric fundus and one in the cardia, all without IM.

*H. pylori, Helicobacter pylori;* IM, intestinal metaplasia; OR, odds ratio; CI, confidence interval; GI, gastrointestinal; GU, gastric ulcer; DU, duodenal ulcer.
